# Analysis of cervical tracheal characteristics of obstructive sleep apnoea-hypopnoea syndrome patients using ultrasound

**DOI:** 10.3389/fmed.2025.1616387

**Published:** 2025-08-19

**Authors:** Haimei Lun, Lisi Wei, Linsong Ye, Liu Wei, Xiaoxi Li, Weixian Zhong, Hongyu Zheng, Qiao Hu

**Affiliations:** ^1^Department of Ultrasound, People's Hospital of Guangxi Zhuang Autonomous Region & Guangxi Academy of Medical Sciences, Nanning, Guangxi, China; ^2^Department of Otolaryngology, Head and Neck, People's Hospital of Guangxi Zhuang Autonomous Region & Guangxi Academy of Medical Sciences, Nanning, Guangxi, China

**Keywords:** cervical trachea, OSAHS, ultrasound, caudal tracheal displacement, breathe

## Abstract

**Importance:**

Cervical tracheal characteristics in OSAHS patients remain unclear.

**Objective:**

This study aimed to analysis the cervical tracheal characteristics of obstructive sleep apnoea-hypopnoea syndrome (OSAHS) patients using ultrasound.

**Methods:**

One hundred and thirteen patients with OSAHS and 113 age-, sex-, height-, weight-, and BMI-matched healthy controls underwent cervical tracheal sonographic examination. Tracheal wall motion was monitored and sonographic measurements of the airway lumen were obtained during quiet respiration, deep respiration, and the Müller maneuver. Tracheal displacement during the Müller maneuver was monitored and quantified. Measurements were compared between groups.

**Results:**

Adequate visualization of cervical tracheal dimensional changes during the Müller maneuver was obtained in 91.15% of patients with OSAHS and 99.12% of healthy subjects. The cervical trachea had the smallest lateral diameter during the Müller maneuver (*P* < 0.001) in patients with OSAHS and healthy controls. The cervical trachea moved caudally during the Müller maneuver, and its peak tracheal displacement was greater in patients with OSAHS than that in the healthy controls (*P* < 0.001). There was a positive relationship between peak tracheal displacement during the Müller maneuver and apnoea-hypopnoea index (AHI; *P* < 0.001).

**Conclusion:**

OSAHS patients have greater caudal tracheal displacement and greater tracheal size than healthy controls. Ultrasonography may be especially suited for precisely evaluating the trachea and can provide a reference for the airway assessment and management of OSAHS.

## Highlights

- Question: How do cervical tracheal features vary in OSAHS patients, and what are their clinical implications?
- Findings: This study found that OSAHS patients exhibit greater caudal tracheal displacement and larger tracheal size compared to healthy controls. Notably, such differentiation must be considered during OSAHS patient airway assessment and management.
- Meaning: Ultrasonography may be especially suited for precisely evaluating the trachea and can provide a reference for airway assessment and management for OSAHS.

## Introduction

The trachea serves as the only gateway between the larynx and lungs, and is involved in phonation, deglutition, and airway protection. Tracheal disorders can usually reduce the free lumen diameter or wall stiffness, and hence, limit airflow. Dynamic changes in the tracheal dimension are the most important in identifying tracheal collapse, which also affects humans (1). Presence of a patent airway is vital for patient survival. Additionally, its anatomy is important when tracheal intubation, stenting, endoscopy, and transplantation are performed. Obstructive sleep apnoea-hypopnoea syndrome (OSAHS) is an increasingly prevalent disease characterized by frequent apnoea and hypopnoea, caused by partial or complete obstruction of the upper airways during sleep (2). Anatomical abnormalities of the upper airway have long been known to contribute to obstructive OSAHS and have been extensively studied. Because the trachea and upper airway are connected and thus they form a complex biomechanical system, functional connections among these anatomical structures need to be taken into account (3). Prior studies have indicated that tracheal displacement may increase pharyngeal collapsibility during sleep and that the trachea contributes to the maintenance of pharyngeal patency during wakefulness in OSAHS patients (4, 5). Ðanić et al. described two patients with severe obstructive sleep apnoea, in whom preoperative drug-induced sleep endoscopy revealed upper airway and cervical tracheal collapse at the level of the previous tracheostomy (6). Thus, we hypothesized that there are differences in tracheal displacement and size between patients with OSAHS and healthy subjects. Furthermore, the evaluation of dynamic tracheal motion not only provides the biomechanics and pathophysiology for OSAHS diagnosis but also helps doctors determine the proper endotracheal tube (ETT) size for airway management in the ICU and operating room for patients with OSAHS. However, to the best of our knowledge, the US has not been applied to assess tracheal movement in individuals with OSAHS. Therefore, this study aimed to analysis the cervical tracheal characteristics of OSAHS patients using ultrasound.

## Materials and methods

Informed consent was obtained from all the patients, and the study protocol was approved by the institutional review board of the hospital (No. KY-LW-2022-5).

### Participants

Participants in this study were categorized into two groups (OSAHS and healthy subjects).

#### OSAHS group

Patients were enrolled in this study if they met the following inclusion criteria: (1) patients who underwent overnight PSG examinations and were diagnosed with OSAHS based on overnight PSG and (2) patients who had not received prior treatment for OSAHS before the ultrasonic examination. The ultrasonic examination was performed the next morning after PSG. The exclusion criteria were as follows: cooperated poorly, definite deformations of head and neck such as retrognathia, history of head and neck surgery and radiotherapy, recent head and neck disease or cancer, history of respiratory diseases other than OSAHS. After applying these criteria, 113 patients were enrolled in this study.

#### Healthy subject group

One hundred and thirteen body mass index (BMI)-, sex-, and age-matched healthy participants agreed to participate in this study. All of them underwent a thorough history and physical examination. All healthy subjects were free of any symptoms suggesting sleep apnea (such as snoring; often feeling tired, fatigued, sleepy during the day; stopping breathing during their sleep; high blood pressure. and so on), other sleep disorders, no history of obstructive upper airway disease, and no history of disease affecting the respiratory system. BMI, sex, and age of healthy subjects were matched with those of patients with OSAHS.

#### PSG

Overnight PSG was performed using a digital EMBLA Titanium™ system (Embla, Broomfield, USA). All suspected patients underwent PSG in the sleep laboratory. PSG included an electroencephalogram (EEG), electro-oculogram, chin electromyogram, electrocardiogram, airflow, chest and abdominal wall movement, snoring, and arterial oxygen saturation (SpO2) according to recommendations from the American Academy of Sleep Medicine (AASM) 2012. Technicians analyzed the sleep states according to the manual. The apnea- hypopnea index (AHI) was calculated as the total number of episodes of apnea and hypopnea per hour of sleep based on the PSG results. OSAHS was defined as AHI > 5 events/h. The lowest oxygen saturation (LSAT) was also recorded for each patient.

### Ultrasonic examination

**Figure 1:**
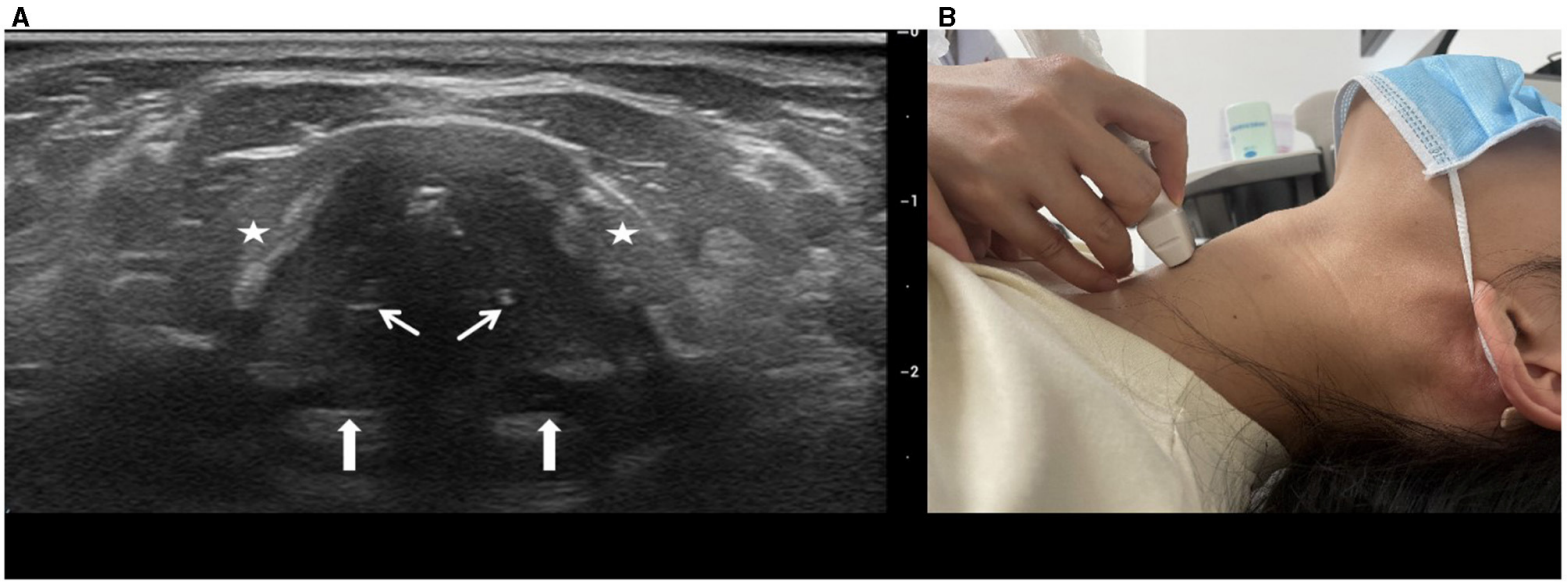
**(A)** Transverse sonograms of true vocal cords during quiet respiration. True vocal cords (small white arrows) appear hypoechoic and stretch from the inner cortex of the thyroid cartilage (asterisk) to the vocal processes of the arytenoid cartilage (large white arrows). **(B)** Transducer position on skin.

**Figure 2:**
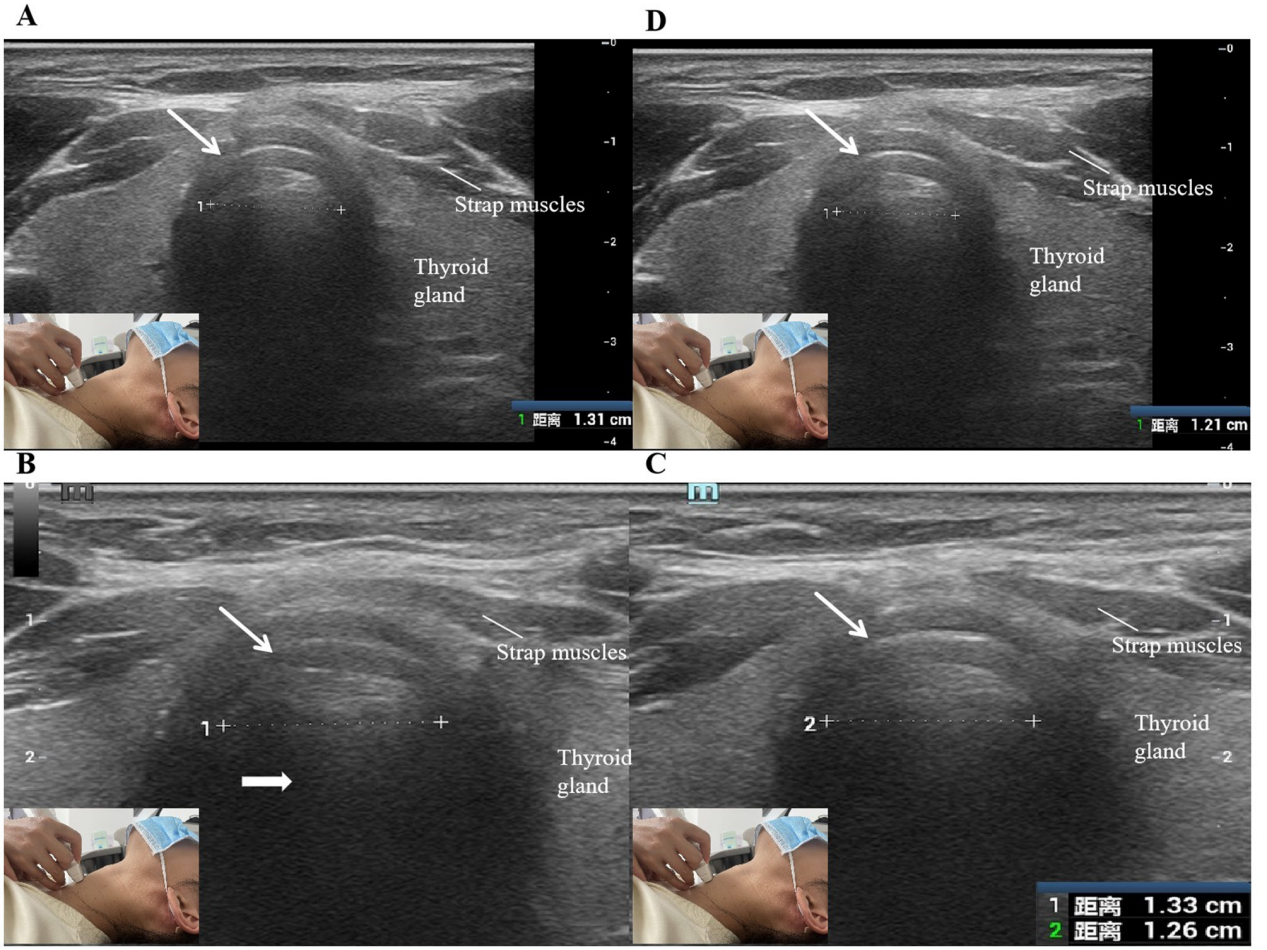
**(A)** Transverse sonograms of the cervical trachea during quiet respiration. **(B)** and **(C)** Transverse sonograms of cervical trachea during deep inspiration and expiration. **(D)** Transverse sonogram of the cervical trachea during Müller maneuver. Tracheal cartilage is a C-shaped hypoechoic structure (small arrows). Air within the cervical trachea produces a trail of strong echoes with characteristic air artifacts (large arrows). 1 and 2, lateral diameters of the cervical trachea (the distance from the inner edge of the right wall of the tracheal cartilage to the inner edge of the left wall). The insets show the transducer position on the skin.

**Figure 3:**
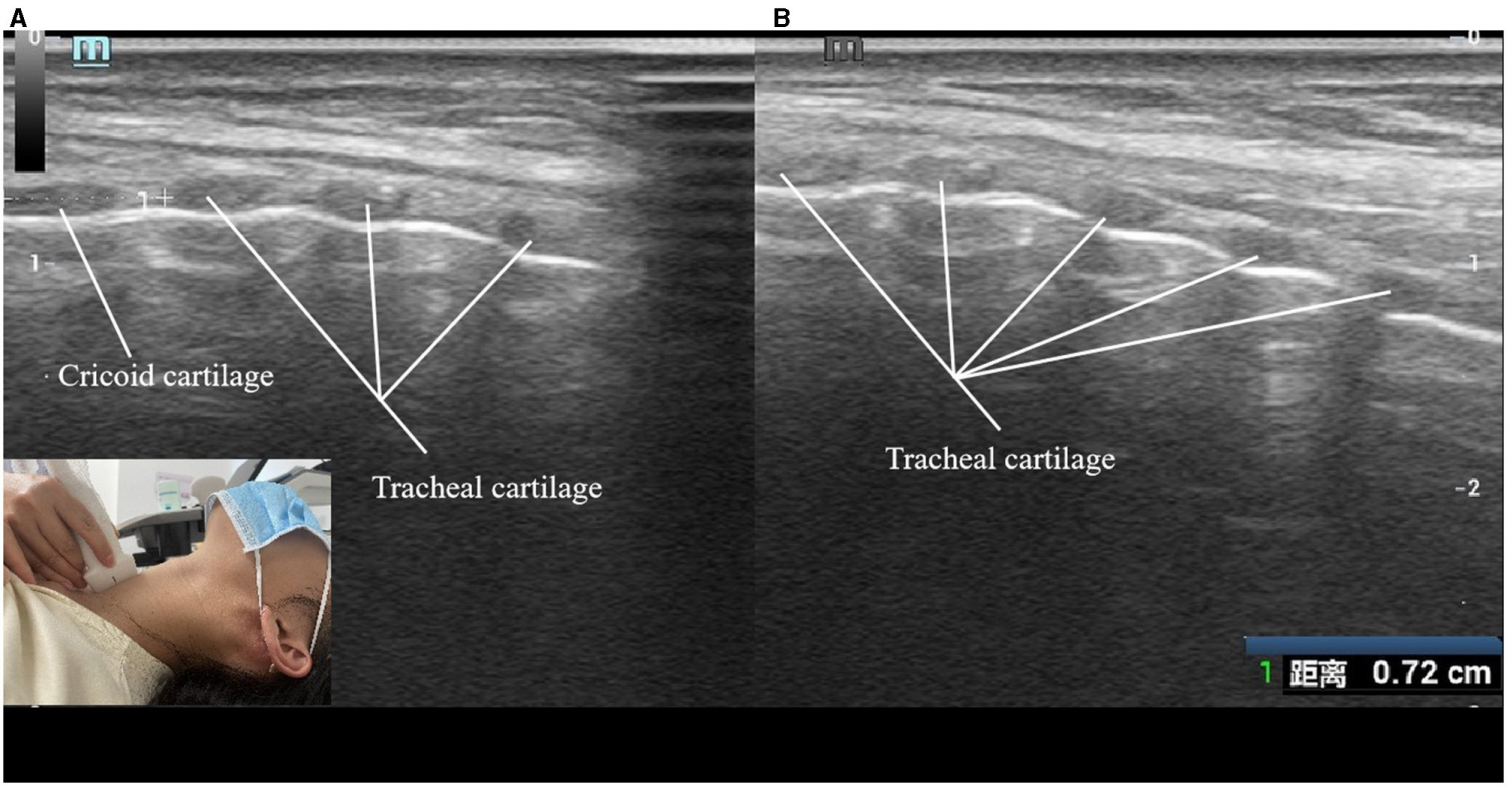
**(A)** Cervical trachea in sagittal plane (during Müller maneuver) **(B)** Cervical trachea in sagittal plane (during quiet respiration). 1, peak caudal tracheal displacement during Müller maneuver. The insets show the transducer position on the skin.

A Resona 70 B device (Mindray Medical International Ltd., Shenzhen, China) with a 6- to 13-MHz linear transducer was used. The participants underwent a sonographic examination in the supine position while they were fully awake. The head and neck were placed in a neutral position with the neck hyperextended. The procedural setup for using a linear transducer to acquire cervical trachea images is as follows:

(1) Transverse view
  The ultrasound transducer was oriented transversely across the anterior surface of the neck. The ultrasonography procedure began with the location of false vocal cords (paired hyperechoic structures; Figure 1). The probe was then moved caudally to visualize the cervical trachea under different breathing conditions, including quiet respiration, deep respiration, and the Müller maneuver. The lateral diameter of the cervical trachea was measured during quiet respiration, end of deep inspiration, end of deep expiration, and the Müller maneuver (Figure 2).
(2) Longitudinal view
  The transducer was placed longitudinally along the anterior median surface of the neck, covering the cricoid cartilage via the cervical tracheal ring (Figure 3). The tracheal cartilage landmarks were manually tracked for the duration of the Müller maneuver. Peak caudal tracheal displacement was quantified as the difference between the most craniad position during quiet respiration and the most caudal position during the Müller maneuver (Figure 3). For analysis, we identified the first cervical tracheal ring as a landmark to track tracheal movement. To maintain uniformity in the measurements, the same operator (author H.M.L. with 12 years of experience) performed the sonographic procedure. To reduce measurement errors, the procedure was repeated three times on three separate images, and the mean value was obtained for analysis.

Reliability and reproducibility studies have also been conducted. Sonographic examinations for interobserver and intraobserver agreement analyses were performed on 40 healthy controls (20 male and 20 female; age range 30–72 years). These examinations were independently performed by two operators (H.M.L. and Q.H.) on the same day to assess interobserver reliability. Operators were blinded to each other during the examination. The participants were scanned by the first operator (H.M.L.) 2 weeks later to determine intraobserver reliability.

### Statistical analysis

Statistical analysis was performed using SPSS software, version 26.0 (SPSS Inc., Chicago, Ill, USA) and GraphPad Prism 8 software. Data are presented as the mean ± standard deviation (SD). Differences in US measurements between quiet respiration and the Müller maneuver, as well as between the end of deep inspiration and the end of deep expiration within the same group, were also calculated using a paired *t*-test. Two-sample *t*-test was used to compare the US measurements among the groups. Independent prediction role of ultrasonic parameters was evaluated through a binary logistic regression method. Receiver operating characteristics (ROC) curves were generated to determine the area under the curve (AUC) to evaluate the ability of the ultrasonic parameters as diagnostic markers. Youden's score was used to detect optimal sensitivity and specificity. Spearman's correlation test was performed to evaluate the association between AHI, LSAT, and US measurements in patients with OSAHS. Data were considered statistically significant when the *P* value was < 0.05.

For reliability and reproducibility studies, intraclass correlation coefficients (ICCs) were used to assess the inter- and intraobserver reliabilities of sonographic measurements of the trachea, with ICC values >0.7 indicating good reproducibility. Good reliability was indicated with values of internal consistency (Cronbach's α) >0.8.

## Results

### Rates of visualization

The visualization rates of the trachea at different breathing statuses were determined according to whether the trachea could be imaged or not. In all participants, the trachea during quiet respiration and deep expiration was easily visualized by sonography, whereas during the Müller maneuver, the trachea was visualized in 91.15% (103 of 113) of patients with OSAHS and 99.12% (112 of 113) of healthy controls. The visualization rate of the trachea during deep inspiration in healthy controls and OSAHS patients were 100% (113 of 113) and 97.35% (110 of 113), respectively.

### Ultrasound imaging

#### Transverse view

The trachea was visible in the transverse view as a cricoid hyperecho with air artifacts posteriorly (Figure 2). The lateral walls of the trachea in patients with OSAHS and healthy controls moved outward, and the lateral dimensions tended to increase during deep inspiration. It reaches its maximum displacement at the end of deep inspiration. The lateral walls of the trachea move inward during deep expiration and the Müller maneuver.

#### Longitudinal view

In the longitudinal section, the trachea resembled a linear hyperecho with posteriorly shaped air artifacts. The trachea moved caudally during deep inspiration and the Müller maneuver (Figure 3).

### Sonographic measurements of cervical trachea

The lateral diameter of the trachea was greater at the end of deep inspiration than at the end of deep expiration (*P* < 0.001) in both the OSAHS and healthy control groups. The cervical trachea had the smallest lateral diameter during the Müller maneuver (*P* < 0.001) in the OSAHS and healthy control groups. Peak tracheal displacement during the Müller maneuver was greater in the OSAHS group than that of healthy controls group (*P* < 0.001; Table 1).

**Table 1:** Characteristics of the participants.

**Parameter**		**OSAHS (*n* = 113)**	**Healthy controls (*n* = 113)**	***x^2^/F***	***P***
Male sex, *n* (%)		63 (79.3%)	61 (84.8%)	0.071	0.789
Age, year		51.32 ± 13.14	48.28 ± 10.23	13.004	0.054
Height, m		1.63 ± 0.08	1.64 ± 0.08	0.437	0.528
Weight, kg		66.29 ± 11.63	64.75 ± 8.16	14.511	0.250
BMI (kg/m^2^)		24.91 ± 3.64	24.16 ± 2.15	23.621	0.059
AHI (events/h)		33.28 ± 23.28	____	____	____
LSAT (%)		76.91 ± 12.23	____	____	____
Lateral diameters of cervical trachea (mm)	Quiet respiration	14.86 ± 3.21	13.09 ± 2.14	12.937	< 0.001
	The end of deep inspiration	15.08 ± 3.34	13.13 ± 2.27	9.333	< 0.001
	The end of deep expiration	14.06 ± 3.13	12.32 ± 2.23	7.487	< 0.001
	Muller maneuver	13.38 ± 2.78	11.81 ± 2.05	12.038	< 0.001
The peak tracheal displacement (Muller maneuver) (mm)		12.80 ± 7.91	5.91 ± 4.07	43.321	< 0.001
Lateral diameters changes during deep respiration		6.57 ± 5.07	6.27 ± 4.35	0.802	0.637
Lateral diameters changes during Muller maneuver		10.34 ± 7.99	9.68 ± 6.54	0.551	0.527

OSAHS, obstructive sleep apnoea-hypopnoea syndrome; BMI, body mass index; AHI, apnoea-hypopnoea index; LSAT, the lowest oxygen saturation.

**Figure 4:**
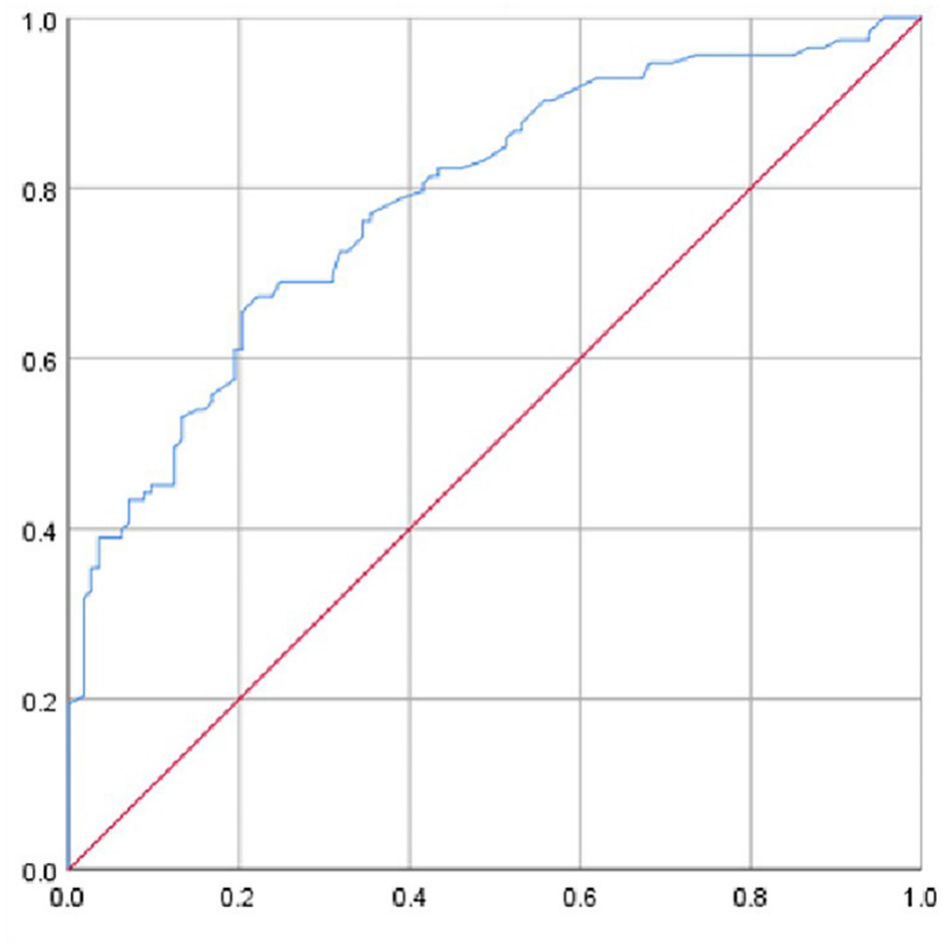
ROC curve of ultrasound quantitative parameters.

In binary logistic regression analysis, peak tracheal displacement during the Müller maneuver was the most important predictive factor for OSAHS (*P* < 0.001; Table 2). ROC curves were applied to determine the sensitivity and specificity of the peak tracheal displacement during the Müller maneuver. As depicted in Figure 4, the AUC value of the peak tracheal displacement during the Müller maneuver was 0.786. A peak tracheal displacement during the Müller maneuver higher than 8.55 mm had the highest diagnostic value for OSAHS, with a sensitivity of 67.3% and a specificity of 77.9%.

**Table 2:** Results of binary logistic regression analysis for OSAHS.

**Parameter**		**Be**	**Crude OR**	**(95%CI)**	***P***
Lateral diameters of cervical trachea	Quiet respiration	0.226	1.25	0.88–1.79	0.215
	The end of deep inspiration	0.340	1.40	0.84–2.35	0.195
	The end of deep expiration	−0.198	0.82	0.49–1.39	0.461
	Muller maneuver	−0.139	0.87	0.60–1.25	0.455
The peak tracheal displacement (Muller maneuver)		0.188	1.21	1.13–1.29	< 0.001

**Figure 5:**
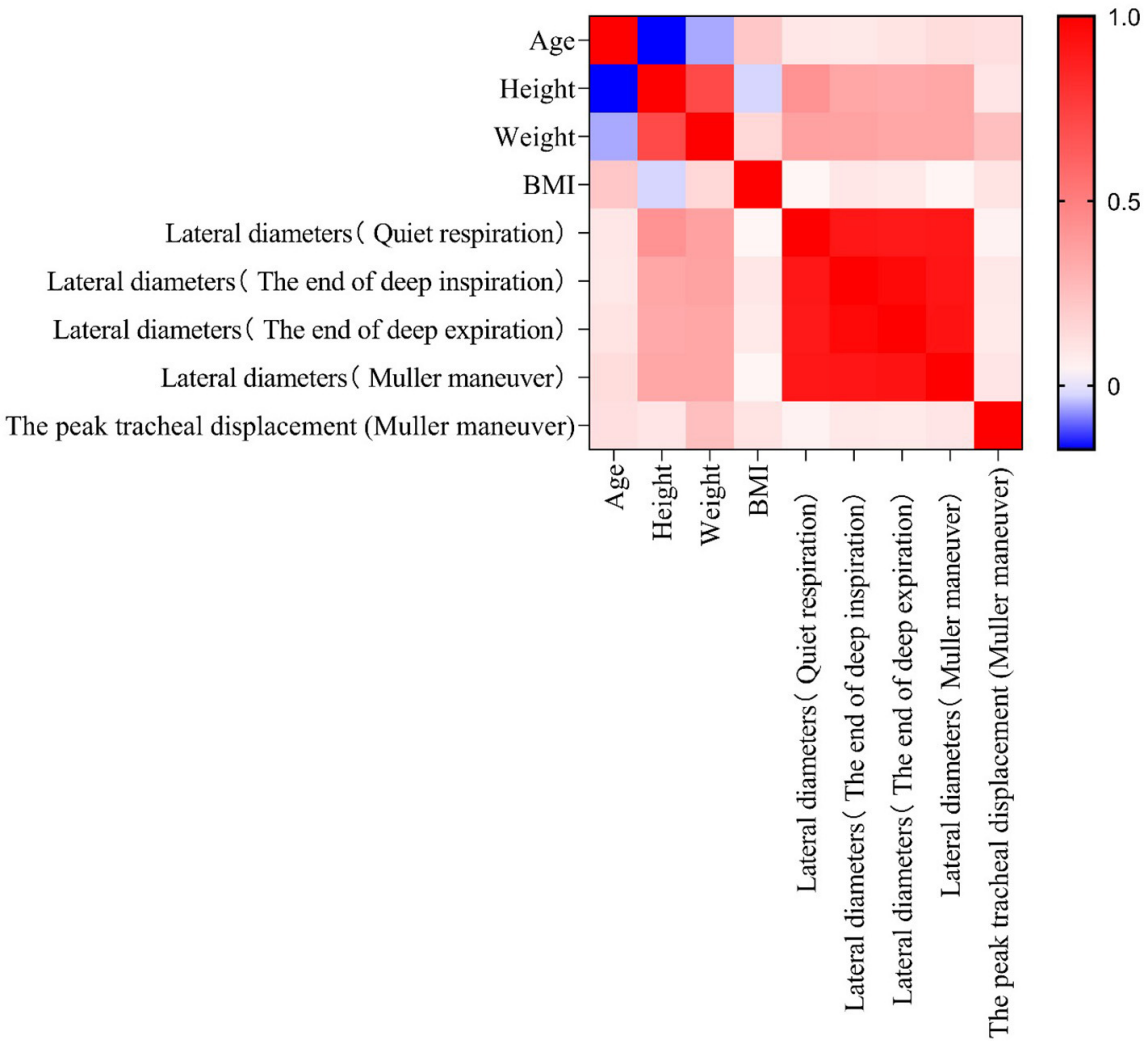
Correlation matrix for all ultrasound measurements, BMI, height, weight, and age in the healthy control group. Red and blue denote the positive and negative correlations, respectively. The darker the color, the higher is the correlation (*P* < 0.05).

**Figure 6:**
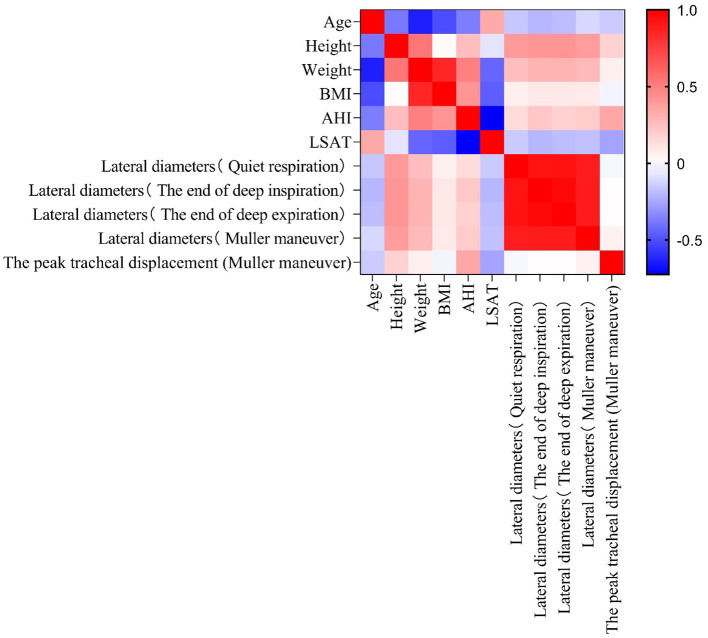
Correlation matrix for all ultrasound measurements, BMI, AHI, LSAT, height, weight, and age in the OSAHS group. Red and blue denote the positive and negative correlations, respectively. The darker the color, the higher is the correlation (*P* < 0.05).

Peak tracheal displacement during the Müller maneuver in patients with OSAHS correlated with an increased AHI (*P* < 0.01). In the OSAHS group, there was no relationship between the peak tracheal displacement during the Müller maneuver and anthropometric parameters (age, height, weight, or BMI; *P* > 0.05). Lateral diameters of the cervical trachea were positively correlated with height and weight but were not associated with BMI in both OSAHS patients and healthy control subjects (*P* < 0.05; Figures 5, 6), respectively.

### Reliability and reproducibility of sonographic measurements

Among the 40 participants who participated in the reliability and reproducibility studies, sonographic measurements of the cervical trachea had higher reliability and reproducibility (0.964–0.987 for interobserver reliability and 0.787–0.975 for intraobserver reliability; Table 3).

**Table 3:** Intraobserver variability and interobserver variability in the sonographic measurements of cervical trachea.

**Parameter**		**Interobserver variability**		**Intraobserver variability**	
		**Cronbach** α	**ICC**	**Cronbach** α	**ICC**
Lateral diameters of cervical trachea	Quiet respiration	0.987	0.975	0.994	0.987
	The end of deep inspiration	0.881	0.787	0.982	0.964
	The end of deep expiration	0.982	0.965	0.987	0.975
	Muller maneuver	0.988	0.975	0.990	0.980
The peak tracheal displacement (Muller maneuver)		0.985	0.970	0.986	0.972

ICC, intraclass correlation coefficient.

## Discussion

The tracheas of the cervical trachea are composed of C-shaped tracheal cartilage that opens dorsally and the tracheal muscle bridges the space. All of these structures were used as acoustic windows to obtain airway sonographic images. In this study, we found that it can be dynamically observed through ultrasound that all these structures move caudally during deep respiration and Müller maneuver. In some cases, particularly in those with severe OSAHS, the cervical trachea almost fully descends into the thoracic cavity at the end of deep inspiration and the Müller maneuver, making it undetectable on ultrasound. As a result, the rates of visualization of the trachea were both lower at the end of deep inspiration and the Müller maneuver than during quiet or deep expiration.

In this study, caudal displacement of the trachea during the Müller maneuver was greater in patients with OSAHS than in the healthy controls. According to recent studies, the degree of tracheal displacement varies directly with lung volume, that is, increased lung volume improves airway collapsibility through caudal traction (7). In addition, previous research has suggested that caudal tracheal traction contributes to improved airway ventilation by lengthening the upper airway and changing the upper airway lumen geometry (8). Finally, snoring implies vibrations of the tissues that produce the sound, and the long-standing tissue vibration may cause local nerve lesions, and then might induce structural changes in airway structure (9). The consequence of the several factors discussed above may explain why, in our sample, patients with OSAHS had greater dynamic caudal tracheal displacement than healthy controls during the Müller maneuver. Furthermore, ROC curve analysis indicated that peak tracheal displacement during the Müller maneuver could effectively identify OSAHS, with cutoff values of peak tracheal displacement = 8.55 mm. Among these parameters, a peak tracheal displacement during the Müller maneuver higher than 8.55 mm had the highest diagnostic value. Additionally, a positive correlation was observed between caudal tracheal displacement and the AHI. Tracheal displacement influences the upper airway by transmission of its load via the thyroid cartilage and its attachments to the hyoid bone, highlighting the importance of assessing caudal tracheal displacement for the possibility of OSAHS. Sonography provides a new and effective reference for the differential diagnosis of OSAHS, especially suitable for distressed area, which had the worst health care. Choosing an appropriate ETT size is important for effective and timely airway management. The cervical tracheal examination, which assesses cervical tracheal airway size, may help in selecting the appropriate ETT size for OSAHS patients. This assessment serves as a predictive factor for significantly reducing the rate of post-intubation complications. Sonography may be a useful measuring tools for choosing ETT size and assisting with airway management of OSAHS.

The present study found that the superficial position of the cervical tracheal structures facilitated the identification of the anatomical structures. Ultrasonography could provide a realistic impression of the process of caudal tracheal movement during quiet respiration, deep respiration, and the Müller maneuver; the lateral walls of the trachea of patients with OSAHS and healthy controls move outward, and the lateral dimension tends to increase during deep inspiration. It reaches its maximum displacement at the end of deep inspiration. These findings are similar to previous reports (10–12). Ciet et al. using cine magnetic resonance imaging (MRI), showed that there was considerable fluctuation in the tracheal dimensions in healthy controls and patients with tracheobron -chomalacia between inspiration and expiration (10). Stern et al. in their study group of healthy humans, used computerized tomography (CT) to identify a 35% change in tracheal cross-sectional area at the level of the aortic arch between maximal inspiration and forced expiration (11, 12). Some investigators have previously reported that ultrasonography can be used to visualize and evaluate the cervical trachea. Although several studies have quantitatively evaluated the lateral diameters of the cervical trachea, few have focused on these aspects in different modes of respiration or in patients with OSAHS. In this study, we found that the lateral diameter of the cervical trachea varied significantly during the different respiratory cycles. Patients with OSAHS have greater tracheal dimensions than healthy controls. This implies that under the same physique condition, patients with OSAHS should choose a greater ETT size than healthy controls. In the present study, reliability (ICC) and internal consistency (Cronbach's α) were good for all measurements. These results are encouraging and support the utility of ultrasonography in clinical and future studies. Moreover, patients with OSAHS had larger lateral diameter changes than healthy subjects, although the difference was not statistically significant. Cervical tracheal dimensions are determined by the gradient between atmospheric and intratracheal pressures. The increased tracheal displacement and muscle hypotonia, which could consequently increase the longitudinal tension of the airway wall and decrease extraluminal tissue pressure, may explain this closure (5).

Another finding of this study was that the lateral diameter of the cervical trachea increased with height and weight in patients with OSAHS and healthy controls. Karmakar and Aljathlany made similar measurements by CT-based measuring tools, they found, as we did, height had a consistent significant association with the airway size (13, 14). It is important to note, that we found that the peak tracheal displacement had a positive correlation with AHI and a negative correlation with the LSAT. Our results were consistent with those reported by Tong et al. who found that tracheal displacements during quiet respiration are larger in individuals with more severe OSAHS, and that increased tracheal displacement may contribute to the maintenance of upper airway patency during wakefulness in OSAHS, particularly in those with severe disease, which is consistent with our results (4). However, it is not clear if peak tracheal displacement is sufficient to accurately predict OSAHS in outpatient ear, nose and throat departments, and further studies are needed.

The major limitation of our study is that we measured airway size in awake subjects. While the strengths of our study were age, sex, height, weight, and BMI-matched in both groups, the range of participants' age, sex, height, weight, and BMI was limited, which may limit the generalizability of our results. Finally, an increase in the number of participants would allow multivariate analyses to examine other anatomical and physiological contributors to caudal tracheal displacement.

## Conclusion

In conclusion, ultrasonography could provide a realistic impression of the process of cervical tracheal movement during deep respiration and the Müller maneuver, and perform quantitative analysis of the changes in the cervical tracheal lumen during deep respiration and the Müller maneuver, as well as tracheal displacement during the Müller maneuver. Even under dynamic conditions, movement of the cervical trachea can be repeatedly observed by a sinologist. Patients with OSAHS have greater caudal tracheal displacement and tracheal size than healthy controls do. Ultrasonography may be especially suited for precisely evaluating the trachea and can provide a reference for the airway assessment and management of OSAHS.

## Data Availability

The original contributions presented in the study are included in the article/Supplementary material, further inquiries can be directed to the corresponding authors.
