# Supplementary material for: Analysis of cervical tracheal characteristics of obstructive sleep apnoea-hypopnoea syndrome patients using ultrasound
Source: Front Med (Lausanne). 2025 Aug 19;12:1616387. doi: 10.3389/fmed.2025.1616387 (PMC12401699; doi:10.3389/fmed.2025.1616387)
Supplement: Supplementary file 1 [file Supplementary_file_1.docx]

## **Supplementary Material**

**Table S1** Results of binary logistic regression analysis for OSAHS

| Parameter | | Be | Crude OR | (95%CI) | *P* |
| --- | --- | --- | --- | --- | --- |
| Lateral diameters  of  cervical  trachea | Quiet respiration | 0.226 | 1.25 | 0.88-1.79 | 0.215 |
|  | The end of deep inspiration | 0.340 | 1.40 | 0.84-2.35 | 0.195 |
|  | The end of deep expiration | -0.198 | 0.82 | 0.49-1.39 | 0.461 |
|  | Muller maneuver | -0.139 | 0.87 | 0.60-1.25 | 0.455 |
| The peak tracheal displacement  (Muller maneuver) | | 0.188 | 1.21 | 1.13-1.29 | <0.001 |

**Table S2** Intraobserver variability and interobserver variability in the sonographic measurements of cervical trachea.

| Parameter | | Interobserver Variability | | Intraobserver Variability | |
| --- | --- | --- | --- | --- | --- |
|  |  | Cronbach α | ICC | Cronbach α | ICC |
| Lateral diameters  of  cervical  trachea | Quiet respiration | 0.987 | 0.975 | 0.994 | 0.987 |
|  | The end of deep inspiration | 0.881 | 0.787 | 0.982 | 0.964 |
|  | The end of deep expiration | 0.982 | 0.965 | 0.987 | 0.975 |
|  | Muller maneuver | 0.988 | 0.975 | 0.990 | 0.980 |
| The peak tracheal displacement  (Muller maneuver) | | 0.985 | 0.970 | 0.986 | 0.972 |

Abbreviations: ICC=intraclass correlation coefficient.
